# Inhibition of Aflatoxin Synthesis in *Aspergillus flavus* by Three Structurally Modified Lentinans

**DOI:** 10.3390/ijms15033860

**Published:** 2014-03-04

**Authors:** Jinyou Ma, Haizhen Mo, Ying Chen, Ding Ding, Liangbin Hu

**Affiliations:** 1 Department of Food Science, Henan Institute of Science & Technology, Xinxiang 453003, Henan, China; E-Mails: marsjy@163.com (J.M.); mohz@163.com (H.M.); yingch0816@163.com (Y.C.); 2 Department of Food Science, Northwest A&F University, Yangling 712100, Shannxi, China; E-Mail: totu0_0@163.com

**Keywords:** lentinan, aflatoxin, structural modification, *Aspergillus flavus*

## Abstract

The chemical properties of β-glucans leading to their inhibition on aflatoxin (AF) production by *Aspergillus flavus* remain unclear. In this study, structurally modified lentinan derivatives were prepared by carboxymethylation, sulfation, and phosphorylation to explore their inhibition activity to AF synthesis. The results demonstrated that inhibitory activity of lentinan decreased at higher or lower concentrations than 200 μg/mL. Compared with lentinan, the sulphated derivatives only performed a reduced optimal inhibition rate at a higher concentration. The phosphorylated derivatives achieved complete inhibition of AF production at 50 μg/mL, but the inhibitory activity was attenuated with an increase of concentration. The minimum concentration of carboxymethylated derivatives to completely inhibit AF synthesis was the same as that of the original lentinan, whereas their inhibition activity was not reduced at the increasing concentration. RT-PCR analyses were conducted to understand the effects of lentinan and its carboxymethylated derivatives on the transcription of certain genes associated with AF biosynthesis. The results showed that lentinan delayed the transcription of aflQ, whereas its carboxymethylated derivatives promoted the transcriptions of all the tested genes. Our results revealed that some chemical group features apart from the β-bond could play the vital role in the prevention of AF formation by polysaccharide, and highlighted the structural modifications which could promote its practicability in the control of aflatoxin contamination.

## 1. Introduction

*Aspergillus flavus* is a fungus able to contaminate food commodities and feeds by producing aflatoxins (AFs) that are carcinogenic, teratogenic, and mutagenic for animals and humans [1]. There are more than 25 enzymatic steps involved in AF biosynthesis [2], and the corresponding genes are clustered in the genome suffering from multiple regulations [3]. For food safety reasons, both the authorities and industries need to control the AF contamination in food and feed below the allowed level (<20 μg/kg, FDA). Many researchers have screened numerous natural products, synthetic compounds, and extracts from diverse organisms for inhibitors of AF biosynthesis [4–6]. Many of them showed different modes of action, and could also be used to identify regulatory networks controlling aflatoxin biosynthesis [4]. Among the screened inhibitors, food-grade products are preferred for application in food and feeds preservation due to their low impact on the environment and on human health [7].

Glucans from mushrooms, which are pharmacologically classified as biological-response modifiers and immunomodulators, have been found to attenuate aflatoxicosis [8]. The β-d-glucans, and specifically (1,3)-β-d-glucans that are moderately branched with (1,6)-β-d-glucan chains from the cell wall of *Saccharomyces cerevisiae*, are capable of adsorbing AFB1 through the formation of hydrogen bonds [9]. Reverberi *et al.* suggested that β-glucans from *Lentinus edodes* could inhibit AF biosynthesis and be a promising tool against AF contamination [7]. Another study demonstrates that the β-glucans from *Trametes versicolor* extracts are also able to inhibit AF production in *A. parasiticus* [10]. Fungal β-glucans have established a close relationship with AF. However, the chemical properties of β-glucans associated with their inhibitory effect on AF production in *A. flavus* have not been deciphered.

**Figure 1.**
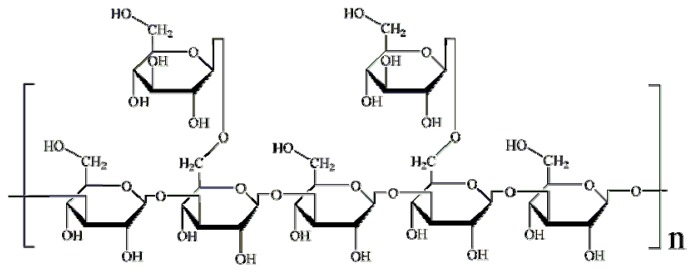
Chemical structure of lentinan.

Lentinans structurally comprise (1,3)-β-d-glucans that are moderately branched with (1,6)-β-d-glucan chains (Figure 1) and have a stimulatory effect on innate and adaptive immunity against tumours and pathogens [11,12]. Although lentinans were suggested with inhibitory activity against AF production in *A. parasiticus* [7], the direct evidence is too limited. In this study, the effects of lentinan and its structural derivatives on the AF production in *A. flavus* were investigated to elucidate the chemical properties of β-glucan associated with the inhibitory activity to AF biosynthesis. In addition, RT-PCR was also used to better understand the potential mechanism leading to aflatoxin reduction.

## 2. Results and Discussion

### 2.1. Results

#### 2.1.1. Structural Modification

**Figure 2.**
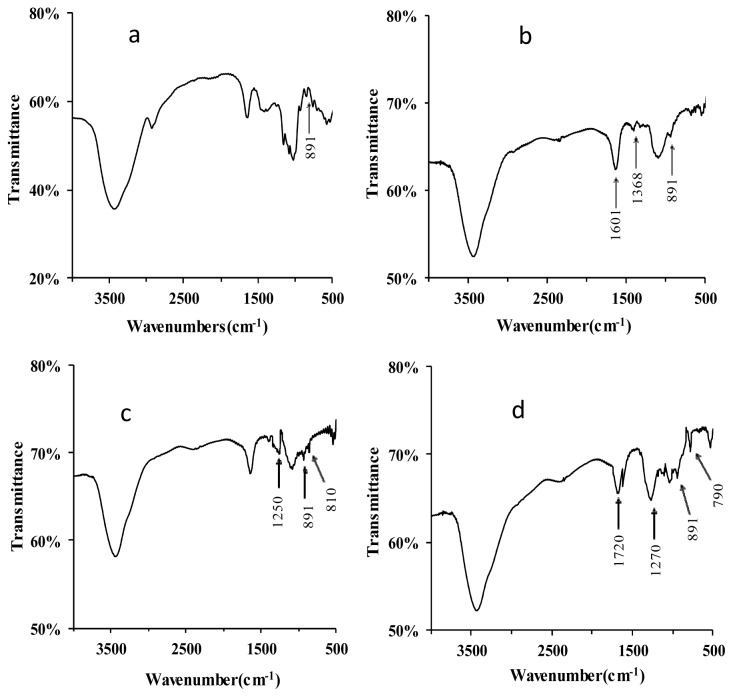
FT-IR spectra of lentinan (**a**); C-D (**b**); S-D (**c**); and P-D (**d**). C-D: carboxymethylated derivatives of lentinan; S-D: sulphated derivatives of lentinan; P-D: phosphorylated derivatives of lentinan.

The FT-IR spectra of lentinan and its derivatives are shown in Figure 2. The native lentinan exhibited two strong absorption bands at 3447 and 1050 cm^−1^ (Figure 2a), which are associated with “O–H” and “C–O” bonding, respectively. Compared to lentinan, two new absorption bands at 1601 and 1338 cm^−1^ appeared in the IR spectrum of C–D (Figure 2b), and these indicate the existence of the functional groups “–COOH” and “–CH2–”, respectively, in the carboxymethylated derivatives [11]. In the IR spectrum of S-D, the appearance of an absorption band at 1250 cm^−1^ is due to the vibrations of “S=O” groups (Figure 2c) [12]. In addition, a “C–O–S” bond would give rise to the FT-IR band observed at 810 cm^−1^ (Figure 2c) [13], indicating that the successful sulfation of lentinan. The phosphorylation of lentinan was also confirmed by FT-IR of P-D. Three new strong absorption peaks appeared in the P-D spectrum at 1720, 1270, and 790 cm^−1^, and these were assigned to “C=O” stretching vibration, “P=O” asymmetric stretching, and “C–O–P” symmetric vibrations, respectively (Figure 2d) [14]. These absorptions indicated that the phosphorylated modification of lentinan was successfully performed. The absorption signals at 891 cm^−1^ were found for all the derivatives, indicating that all of these derivatives contained the β-glycosidic linkage of d-glycopyranoside [15].

#### 2.1.2. Effects on Mycelia Growth and Aflatoxin Synthesis

As shown in Table 1, lentinan and its derivatives did not significantly inhibit mycelial growth even at the highest concentration of 600 μg/mL (*p* < 0.05), and none of the three structurally modified lentinans resulted in any significant changes in the mycelial growth of *A. flavus*.

**Figure 3.**
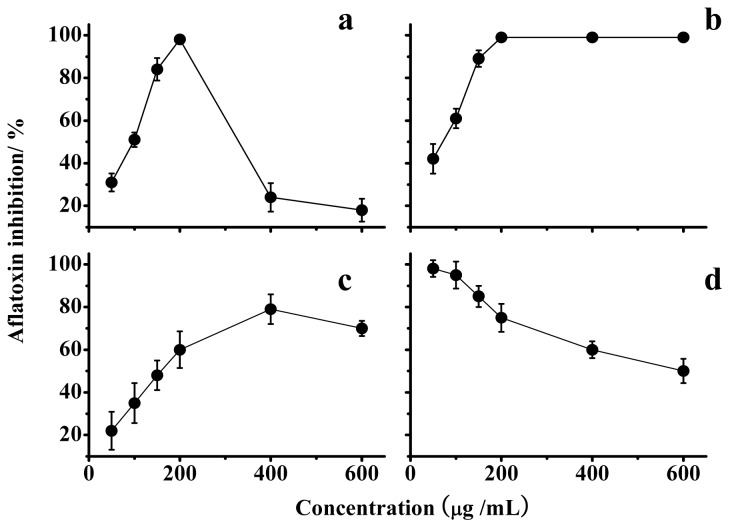
Inhibition of aflatoxin production by lentinan (**a**); C-D (**b**); S-D (**c**); and P-D (**d**). The values are the mean of three replicates ± standard error.

**Figure 4.**
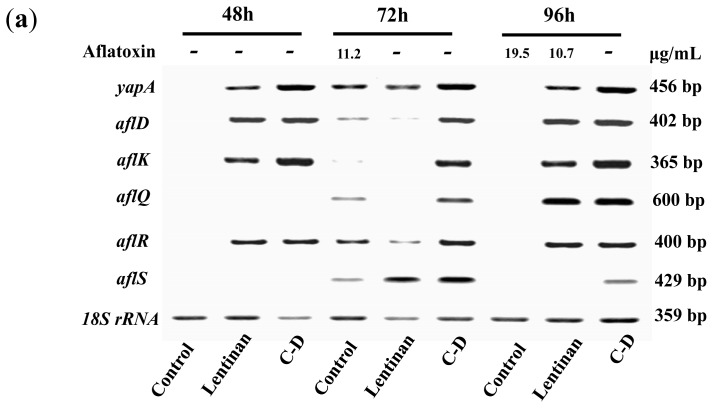

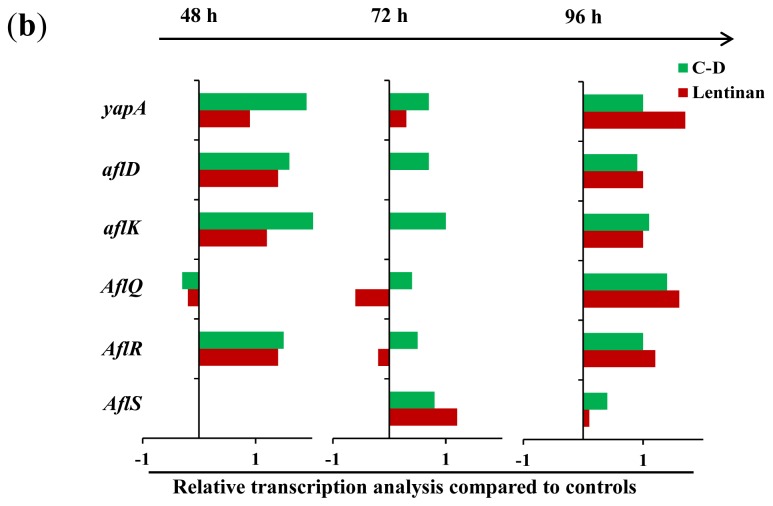
The transcription levels of some genes associated with aflatoxin biosynthesis in *A. flavus*. (**a**) The PCR products in the agarose gel, and their sizes were shown at the right; “−”: no detectable aflatoxin; (**b**) The relative gene transcription normalized on 18S rRNA level compared to control were reported. The relative abundance of the transcripts were obtained by dividing the band intensity of target gene by the band density of the corresponding 18S rRNA. The value of column equals the relative transcription of treatment minus that of control.

Data in Figure 3a indicate that a lentinan concentration of 200 μg/mL resulted in the maximal inhibition. In addition, the optimal inhibition by lentinan was temporary and could be overcome over time (Figure 4a). The chemical modifications of lentinan resulted in unexpected changes in the inhibition of the AF production. Among the lentinan derivatives tested, the optimal inhibition rate of S-D was reduced, and its corresponding concentration also increased compared with lentinan (Figure 3c). P-D and C-D exhibited the same optimal inhibition rate as lentinan, but the concentration-dependent trends were different (Figure 3b,d). P-D could achieve complete inhibition of AF production at a concentration of 50 μg/mL, and this inhibition was attenuated with an increase in the concentration of P-D (Figure 3d). The lowest concentration of C-D that achieved the complete inhibition of AF production was the same as that observed for lentinan, and this inhibition was not changed with an increase in the concentration of C-D (Figure 3b). This stable inhibition aroused our interest in the mechanism through which C-D inhibits AF production.

#### 2.1.3. Gene Transcription Analysis

To understand the mechanism through which these β-glucans exert their inhibitory effects, we analyzed the transcription of certain genes that are known to be associated with AF production in *A. flavus*in response to lentinan and C-D. *ApyapA* orthologs are known to be part of the antioxidant response in fungi and have been found to be associated with AF biosynthesis through the modulation of the reactive oxygen species (ROS) in *A. parasiticus* [16]. It was found that exposure to lentinan and C-D at the inhibitory concentrations led to an earlier, stronger, and longer transcription of the *yapA* orthologue compared to its transcription during the initial stage of AF formation in the control (Figure 4). The comparison of lentinan and C-D revealed that the latter promoted the transcription of *yapA* orthologue at a higher level (Figure 4a,b). Two genes, *aflR* and *aflS*, are involved in the genetic regulation of AF biosynthesis [3]. AflR is required to activate expression of the AF biosynthetic gene cluster [17], whereas AflS modulates the AF expression through its interaction with AflR [18]. It was surprising to find that both lentinan and C-D promoted the transcription of *aflS* and *aflR* in *A. flavus* at an AF-production-inhibitory state (Figure 4a,b). Similar results were found in the transcription of the AF-biosynthesis-related genes *aflD* and *aflK.* Both of these genes were only slightly transcribed 72 h post-incubation (the early stage of AF formation) in the control. However, their transcription was continuously activated in *A. flavus* exposed to lentinan and C-D (Figure 4). It is notable that the treatment with lentinan delayed the transcription of *aflQ* (a gene downstream of the AF biosynthesis pathway) compared with the control, whereas the treatment with C-D promoted *aflQ* transcription at a higher level.

### 2.2. Discussion

In this study, definite effects of lentinan on AF production were revealed. First, the valid inhibitory concentrations were restricted to a narrow range, and the inhibition was only temporary. The AF formation paralleled *aflQ* transcription in *A. flavus* exposed to lentinan and exhibited a common lag time compared with the control. This finding implicated *aflQ* in the lentinan-induced inhibition of AF production. Both lentinan and C-D promoted *aflR* transcription, which is contrary to the downregulation of *aflR* transcription observed in *A. parasiticus* exposed to the β-glucan extract from *L. edodes* [7,19]. All of the derivatives of lentinan tested in this study maintained the β-linkage in their structure but exhibited different inhibition modes against AF formation. Thus, some chemical group features apart from the β-bond could play the vital role in the prevention of AF formation by polysaccharide.

It has been reported that β-glucans of similar structure, molecular weight, and solution conformation exhibit vastly differing biological activities *in vitro* and *in vivo* [9]. In this study, the structural modifications of lentinan also resulted in great changes in the inhibition modes against AF production. In particular, the C-D derivatives possessed a new dose-dependent inhibitory effect toward AF biosynthesis. The biological activities of β-glucans are likely to involve their specific interaction with one or more cell-surface receptors [9,20]. This hypothesis could also partly interpret the “ridge type” dose-dependent inhibition curve by lentinan, S-D and P-D. The binding of these polysaccharides with their receptors occurs only when their affinity is more than the repulsion by the membrane due to the same negative charge of these polysaccharides as membrane. Carboxymethylation of lentinan usually occurs at the “C-2” and “C-4”, which is different from the “C-6” where sulfation and phosphorylation occurs [21,22]. These structural changes might induce a novel recognition by a special receptor, leading to the special inhibition manner of C-D. It is worth making intensive efforts to identify the β-glucan receptors in *A. flavus* because this information will greatly contribute to our understanding of the relationship between β-glucans and AF formation.

There is a close relationship between oxidative stress and AF production [16,23]. The anti-oxidant response transcription factor Yap1 has an apparent role in the regulation of AF biosynthesis [16]. Exposure to lentinan or C-D could lead to a higher transcription level of Yap1 in *A. flavus*, which might result in the inhibition of AF formation well. However, AP-1 (Yap1 othologue) is found in the promoter region of *aflR* and is hypothesised to attenuate the expression of *aflR* [16]. The up-regulation of *aflR* transcription by lentinan and C-D illustrated that the regulation of *aflR* transcription by Ap1 is subject to other factors. It is notable that C-D promoted the transcription of all of the tested AF biosynthesis-related genes, which was inconsistent with its inhibitory effects on AF formation. This phenomenon deserves further investigation.

## 3. Experimental Section

### 3.1. Culture of *A. flavus*

*Aspergillus flavus* CGMCC3.2890 was purchased from the China General Microbial Culture Collection Centre. The strain was cultured on Sabouraud Dextrose (SD) medium containing 4% (*w*/*v*) glucose, 1% peptone, and 2% agar for 3 days. Then spore suspension was made by slightly shaking the plate after the addition of 0.05% Triton X-100 and then counted by blood counting chamber under microscope. Lentinan and its derivatives were added to the SD liquid medium (without agar) at the indicated concentration (25, 50, 100, 200, 400 and 600 μg/mL, respectively) before the inoculation of the spores of *A. flavus* (approximately 10^5^/mL). The inocula were incubated at 28 °C and 120 rpm for 72 h. The AFB_1_ in the culture was determined and compared to that in the control (without tested fractions). The fungal mycelia were also collected after the passing through two layers of cheesecloth. After washing five times with sterile water, the collected mycelia were dried in an oven at 80 °C for the determination of the dry weight.

### 3.2. Aflatoxin Assay

Aflatoxin B_1_ in *A. flavus* culture was extracted with chloroform in a ratio (*v*/*v*) of 1:3 (culture broth/chloroform). The extracts were dried with a nitrogen-blowing instrument (DN-12A, Dongkang Co. Ltd., Tianjin, China), followed by dissolving in methanol, and then filtered through a 0.22 μm microporous membrane for subsequent HPLC analysis. The samples were injected through an Agilent HPLC 1100 system equipped with an ODS column (Cosmosil5C18-AR, 250 × 4.6 mm column, Nacalai tesque, Japan) and maintained at 22 °C. The mobile phase was acetonitrile/methanol/water (1:1:2, *v*/*v*/*v*) at a flow rate of 1 mL/min. The column eluent passed through a variable wavelength detector operated at 365 nm and calculated against the aflatoxin B_1_ standard curve (detection limit, 1.5 μg/L) [24].

### 3.3. Structural Modifications of Lentinan

#### 3.3.1. Carboxymethylation

Lentinan was carboxylated according to the protocol described by Chen *et al.* [11]. Lentinan (0.5 g, purity 98%, Luopukang Medicine Group Co. Ltd., Hangzhou, China), 10 mL of 20% NaOH, and 25 mL of isopropanol were completely mixed in an ice bath under stirring for 3 h. Then 5.25 g of chloroacetic acid, 10 mL of 20% NaOH, and 25 mL of isopropanol were added to the above solution. The final mixture was maintained at room temperature for 3 h and then at 60 °C for 1.5 h. After cooling down, the pH was adjusted to 7.0 by using 0.5 M HCl. After that, the solution was dialyzed by using a dialysis tube with molecular weight cut-off of 8.0 kDa followed by lyophilization to obtain the final carboxymethylated derivatives.

#### 3.3.2. Sulfation

According to the protocol described in a previous study [13], lentinan (1.5 g) was treated with formamide (15 mL) and chlorosulfonic acid (10 mL) at 80–90 °C for 4 h. Propylene oxide (50 mL) was then added to the mixture. The precipitate was resuspended in distilled water, and the pH was adjusted to 10.0–11.0. The products were dialyzed against distilled water by using a dialysis tube with molecular weight cut-off of 8.0 kDa for 24 h, and then lyophilized to obtain the sulphated derivatives.

#### 3.3.3. Phosphorylation

Lentinan was phosphorylated following the procedure described by Wei *et al.* [14]. Lentinan (0.5 g) was suspended in anhydrous *N*,*N*-Dimethylformamide (DMF) (20 mL) at room temperature under stirring for 30 min. Then, 3-phosphonopropionic acid (2.0 g), *N*,*N*′-Dicyclocarbodiimide (2.7 g), and 4-Dimethylaminopyridine (DMAP) (3.1 g) were added, and the mixture was allowed to react for 12 h. The mixture was dialyzed against distilled water with a dialysis tube with molecular weight cut off of 8.0 kDa for 36 h to remove DMF, DMAP, and other potential degradation products. Phosphorylated derivatives of lentinan were obtained after freeze-drying.

### 3.4. Fourier Transform Infrared (FT-IR) Spectra

The derivatives of lentinan were structurally characterized with a Bruker Tensor 27 FT-IR spectrophotometer (BRUKER, Ettlingen, Germany). Five milligrams of the derivatives were ground with KBr powder and then pressed into pellets for FT-IR measurement at the frequency range of 400 to 4000 cm^−1^.

### 3.5. Semi-Quantitative RT-PCR Analysis

Mycelia of *A. flavus* were sampled at 48, 72, and 96 h post-inoculation and stored in liquid nitrogen. The total RNA was extracted with an RNApure Plant Kit (Cat No. CW0559, Beijing CoWin Bioscience Co. Ltd., Beijing, China) according to the manufacturer’s instructions. The extracted RNA was treated with DNase I (Takara, Otsu, Japan) at 37 °C for 30 min to remove the genomic DNA and then reverse-transcribed to form the first-strand cDNA. The reaction was followed by denaturation at 92 °C for 5 min and then cooling to 5 °C. The obtained cDNA was then amplified for semi-quantitative analysis. The primers of *aflR* were designed by Somashekar *et al.* [25], and the other primers were designed by Primer Premier 5.0 (PREMIER Biosoft, Palo Alto, CA, USA). The 18S rRNA gene was used as the internal standard. All of the primers are listed in Table 2. The PCR programmes included 30 cycles of 95 °C for 30 s, annealing for 20 s (46 °C for *aflS*, 57 °C for *aflR*, 49 °C for *aflK*, 58 °C for *aflQ*, 49 °C for *yap*, 49 °C for *aflD*, and 55 °C for 18S *rRNA*), and extension at 72 °C for 1 min. The RNA samples were tested for genomic DNA contamination with the extracted RNA directly as the PCR template prior to cDNA synthesis and under the same PCR conditions. The RT-PCR products were separated on 2% agarose gels and stained with ethidium bromide. All of the RT-PCR reactions were performed at least three times. Densitometric scanning using a computer-assisted image analysis system (IP Lab Gel; Signal Analytics Corp., Vienna, VA, USA) was used to quantify the signal intensity of each band. The relative abundance of the transcripts were obtained by dividing the band intensity of target gene by the band density of the corresponding 18S rRNA.

### 3.6. Statistics

All the data presented are the mean value ± standard errors of the means (SEM) of three determinations. One-way ANOVA was used to determine whether there are significant differences between the means in all of the experiments. The differences were considered to be significant if the *p* value was less than 0.05.

## 4. Conclusions

Of the three structurally modified lentinans, only C-D exhibited an acceptable dose-dependent activity in the prevention of aflatoxin generation. Our results revealed that some group features besides the β-bond should be considered in the activity of glucans inhibiting AF synthesis, and carboxymethylation modification could be applied in the development of glucan agents against aflatoxin contamination.

## Figures and Tables

**Table 1.** The effects of different concentrations of lentinan and its derivatives on the mycelial growth of *A. flavus*.

Compounds	Mycelial weight (g)					

	0 (control)	50 μg/mL	100 μg/mL	200 μg/mL	400 μg/mL	600 μg/mL
*lentinan*	0.176 ± 0.025	0.173 ± 0.013	0.145 ± 0.015	0.162 ± 0.030	0.187 ± 0.026	0.212 ± 0.024
C-D	--	0.157 ± 0.022	0.197 ± 0.030	0.182 ± 0.019	0.208 ± 0.020	0.170 ± 0.006
S-D	--	0.145 ± 0.022	0.152 ± 0.023	0.166 ± 0.018	0.202 ± 0.021	0.193 ± 0.005
P-D	--	0.149 ± 0.026	0.176 ± 0.021	0.181 ± 0.021	0.190 ± 0.027	0.212 ± 0.087

The values are the mean of three replicates ± standard error. The means that are not followed by a letter are not significantly different (*p* > 0.05) according to Tukey’s HSD.

**Table 2.** Gene-specific primer pairs used for RT-PCR.

Gene	Primer sequence
*yap*	Forward 5′-TGCAACCTCTCTACAAGCCG-3′ Reverse 5′-CCGAAGTCTCGAGAAAGAGCC-3′
*aflK*	Forward 5′-GAACTGCTTCAGTTGCCGTG-3′ Reverse 5′-ACGAGGGTTCGTTTCTGGAC-3′
*aflD*	Forward 5′-TCCAGGCACACATGATGGTC-3′ Reverse 5′-TGTGGATAACGAAGTGCCCC-3′
*aflQ*	Forward 5′-TTAAGGCAGCGGAATACAAG-3′ Reverse 5′-GACGCCCAAAGCCGAACACAAA-3′
*aflR*	Forward 5′-GCACCCTGTCTTCCCTAACA-3′ Reverse 5′-ACGACCATGCTCAGCAAGTA-3′
*aflS*	Forward 5′-GGAATGGGATGGAGATG-3′ Reverse 5′-GGAATATGGCTGTAGGAAG-3′
*18S rDNA*	Forward 5′-ATGGCCGTTCTTAGTTGGTG-3′ Reverse 5′-GTACAAAGGGCAGGGACGTA-3′

## References

[1] Wagan N.G. (1992). Aflatoxins as risk factors for hepatocellula carcinoma in huamans. Cancer Res..

[2] Yu J.J., Chang P.K., Ehrlich K.C., Cary J.W., Bhatnagar D., Cleveland T.E., Payne G.A., Linz J.E., Woloshuk C.P., Bennett J.W. (2004). Clustered pathway genes in aflatoxin biosynthesis. Appl. Environ. Microbiol..

[3] Georgianna D.R., Payne G.A. (2009). Genetic regulation of aflatoxin biosynthesis: From gene to genome. Fungal Genet. Biol.

[4] Holmes R.A., Boston R.S., Payne G.A. (2008). Diverse inhibitors of aflatoxin biosynthesis. Appl. Microbiol. Biotechnol.

[5] Rasooli I., Fakoor M.H., Yadegarinia D., Gachkar L., Allameh A., Rezaei M.B. (2008). Antimycotoxigenic characteristics of *Rosmarinus officinalis* and *Trachyspermum copticum* L essential oils. Int. J. Food Microbiol..

[6] Zhang T., Shi Z.Q., Hu L.B., Cheng L.G., Wang F. (2007). Antifungal compounds from *Bacillus subtilis* B-FS06 inhibiting the growth of *Aspergillus flavus*. World J. Microbiol. Biotechnol..

[7] Reverberi M., Fabbri A.A., Zjalic S., Ricelli A., Punelli F., Fanelli C. (2005). Antioxidant enzymes stimulation in *Aspergillus parasiticus* by *Lentinus edodes* inhibits aflatoxin production. Appl. Microbiol. Biotechnol..

[8] Yogeswari R., Murugesan S., Jagadeeswaran A. (2012). Hepatoprotective effect of oyster mushroom (*Pleurotus Sajor Caju*) in broilers fed aflatoxin. Int. J. Vet. Sci..

[9] Akramiene D., Kondrotas A., Didziapetriene J., Kevelaitis E. (2007). Effects of beta-glucans on the immune system. Medicina.

[10] Li G., He D., Qian Y., Guan B., Gao S., Cui Y., Yokoyama K., Wang L. (2011). Fungus-mediated green synthesis of silver nanoparticles using *Aspergillus terreus*. Int. J. Mol. Sci..

[11] Chen X., Zhang L., Cheung P.C.K. (2010). Immunopotentiation and anti-tumor activity of carboxymethylated-sulfated β-(1→3)-d-glucan from *Poria cocos*. Int. Immunopharmacol..

[12] Chang Y.J., Lee S., Yoo M.A., Lee H.G. (2006). Structural and biological characterization of sulfated-derivatized oat β-glucan. J. Agric. Food Chem..

[13] Huang R., Du Y., Yang J., Fan L. (2003). Influence of functional groups on the *in vitro* anticoagulant activity of chitosan sulfate. Carbohydr. Res..

[14] Wei D., Cheng W., Wei Y., Zhang L. (2012). Phosphorylated modification and *in vitro* antioxidant activity of Radix Hedysari polysaccharide. Glycoconj. J..

[15] Liu J., Sun Y., Yu H., Zhang C., Yue L., Yang X., Wang L., Liu J. (2012). Purification and identification of one glucan from golden oyster mushroom (*Pleurotus citrinopileatus* (Fr) Singer). Carbohydr. Polym..

[16] Reverberi M., Zjalic S., Ricelli A., Punelli F., Camera E., Fabbri C., Picardo M., Fanelli C., Fabbri A.A. (2008). Modulation of antioxidant defense in *Aspergillus parasiticus* is involved in aflatoxin biosynthesis: A role for the ApyapA gene. Eukaryot. Cell.

[17] Price M.S., Yu J., Nierman W.C., Kim H.S., Pritchard B., Jacobus C.A., Bhatnagar D., Cleveland T.E., Payne G.A. (2006). The aflatoxin pathway regulator AflR induces gene transcription inside and outside of the aflatoxin biosynthetic cluster. FEMS Microbiol. Lett..

[18] Chang P.K. (2003). The *Aspergillus parasiticus* protein AFLJ interacts with the aflatoxin pathway-specific regulator AFLR. Mol. Genet. Genomics.

[19] Reverberi M., Zjalic S., Ricelli A., Fabbri A.A., Fanelli C. (2006). Oxidant/antioxidant balance in *Aspergillus parasiticus* affects aflatoxin biosynthesis. Mycotoxin Res..

[20] Nelson E.D., Ramberg J.E., Best T., Sinnott R.A. (2012). Neurologic effects of exogenous saccharides: A review of controlled human animal and *in vitro* studies. Nutr. Neurosci..

[21] Huang X.Y., Kong X.F., Wang D.Y., Hu Y.L. (2007). Research progress on sulfating modification of polysaccharides and sulfated polysaccharides. Chin. Nat. Prod. Res. Dev..

[22] Suárez E.R., Kralovec J.A., Grindley T.B. (2010). Isolation of phosphorylated polysaccharides from algae: The immunostimulatory principle of *Chlorella pyrenoidosa*. Carbohydr. Res..

[23] Yin W.B., Reinke A.W., Szilágyi M., Emri T., Chiang Y.M., Keating A.E., Pócsi I., Wang C.C.C., Keller N.P. (2013). bZIP transcription factors affecting secondary metabolism sexual development and stress responses in *Aspergillus nidulans*. Microbiology.

[24] Alberts J.F., Engelbrecht Y., Steyn P.S., Holzapfel W.H., van-Zyl W.H. (2006). Biological degradation of aflatoxin B1 by *Rhodococcus erythropolis* cultures. Int. J. Food. Microbial..

[25] Somashekar D., Rati E.R., Chandrashekar A. (2004). PCR-restriction fragment length analysis of aflR gene for differentiation and detection of *Aspergillus flavus* and *Aspergillus parasiticus*in maize. Int. J. Food Microbiol..

